# A child with fever, cough and Lancisi’s sign

**DOI:** 10.11604/pamj.2018.30.14.15397

**Published:** 2018-05-08

**Authors:** Antoine Lamblin, Clément Derkenne

**Affiliations:** 1 Anesthesia-Resuscitation Department, Percy Army Training Hospital, Clamart, France; 2 Paris Fire Department, Paris, France

**Keywords:** Lancisi´s sign, endocarditis, septic pulmonary emboli

## Image in medicine

An 8-year-old boy from a rural town in Chad was referred to the emergency department following a one-month history of fever, otitis media and left thigh osteoarthritis. Venous jugular examination showed prominent systolic pulsations, also called Lancisi's sign. A chest X-ray (A) showed a right pneumothorax, which was immediately drained, before incision and drainage of the left distal thigh osteoarthritis. A CT-scan showed multiple pulmonary abcesses (B). After transthoracic echocardiography (M-TurboTM, Sonosite^®^ Inc, Bothwell, WA, USA), large vegetations were seen on the tricuspid valve (C) and right ventricular systolic pressure was elevated. There was severe tricuspid regurgitation. Microbiological analysis found methicillin-sensitive Staphylococcus aureus in blood cultures and bacteriological samples from the thigh. This case demonstrates a presentation of right-sided endocarditis with septic pulmonary emboli (SPE) responsible for a right pneumothorax. Prominent systolic pulsations of the jugular veins, or Lancisi's sign, are a manifestation of severe tricuspid regurgitation. They result from a retrograde blood flow in the right atrium due to tricuspid incompetence during systole. Pneumothorax, secondary to SPE, is rare and Staphylococci are the infectious pathogens in all reported cases of secondary pneumothorax due to SPE, characterized by pulmonary inflammation with peripheral necrosis and multiple small cavities which involves the pleura; it may progress into the subpleural tissues or fistulize in situ. In this case, targeted antibacterial therapy was initiated until day 42. A rapid clinical improvement was observed. A follow-up transthoracic echocardiograph was performed at 3 months and revealed a reduction in vegetative growth and mild tricuspid regurgitation sequela.

**Figure 1:**
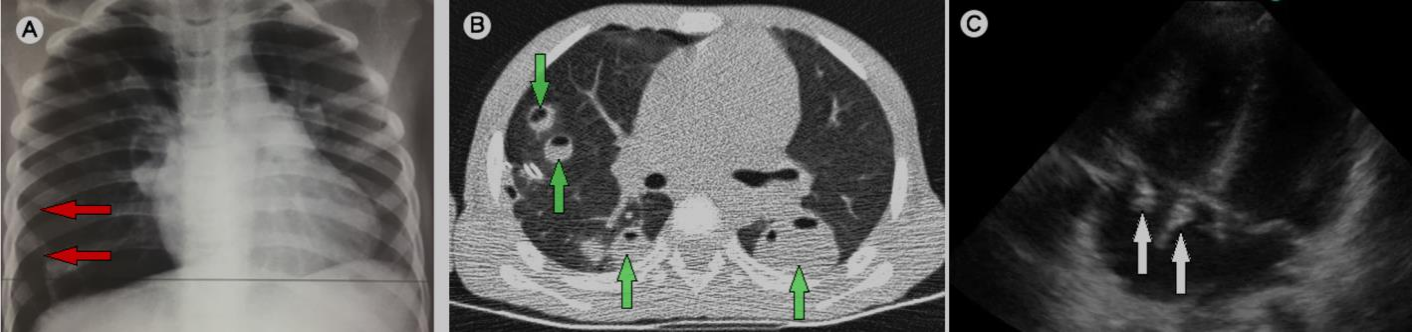
Imaging findings in a right-sided endocarditis with spontaneous pneumothorax and septic pulmonary emboli: (A) chest radiograph at admission, showing a right-sided hemithoracic pneumothorax (red arrows) and a partially-collapsed right lung; (B) lung CT scan on day 1: multiple, differently-sized nodules with cavities in the left and right lungs (green arrows) indicative of septic pulmonary emboli; (C) an apical four-chamber-view transthoracic echocardiograph showing tricuspid valve vegetations in the right atrium during systole (white arrows)

